# Pathway analysis from lists of microRNAs: common pitfalls and alternative strategy

**DOI:** 10.1093/nar/gkv249

**Published:** 2015-03-23

**Authors:** Patrice Godard, Jonathan van Eyll

**Affiliations:** 1 IP & Science, Thomson Reuters, 5901 Priestly Drive, #200, Carlsbad, CA 92008, USA; 2 UCB Pharma, Chemin du Foriest, 1420 Braine-l'Alleud, Belgium

## Abstract

MicroRNAs (miRNAs) are involved in the regulation of gene expression at a post-transcriptional level. As such, monitoring miRNA expression has been increasingly used to assess their role in regulatory mechanisms of biological processes. In large scale studies, once miRNAs of interest have been identified, the target genes they regulate are often inferred using algorithms or databases. A pathway analysis is then often performed in order to generate hypotheses about the relevant biological functions controlled by the miRNA signature. Here we show that the method widely used in scientific literature to identify these pathways is biased and leads to inaccurate results. In addition to describing the bias and its origin we present an alternative strategy to identify potential biological functions specifically impacted by a miRNA signature. More generally, our study exemplifies the crucial need of relevant negative controls when developing, and using, bioinformatics methods.

## INTRODUCTION

MicroRNAs (miRNAs) are small non-coding RNA (∼22 nt) involved in the post-transcriptional regulation of gene expression. miRNAs promote the degradation or inhibit the expression of messenger RNA by binding to specific sequences generally located in the 3′ UTR of their target (1). Therefore, miRNAs can impact the expression of hundreds of genes and are important regulators of biological processes. As such profiling their expression is insightful and has been applied to many organisms and conditions (2).

In order to interpret the biological impact of the miRNAs associated to a condition, studies often include an *in silico* analysis of pathways based on the known or inferred miRNA target genes. In human, for example, miRNA signatures of different diseases such as cancer (3), diabetes (4), infectious disease (5) or various neurodegenerative disorders (6–10) have been described along with hypotheses about the biological processes they ultimately regulate.

Here we show that the *in silico* approach widely applied in such studies, to identify pathways regulated by miRNA signatures, is strongly biased and always leads to the identification of highly-related biological processes. We also explore alternatives to this approach, deliberately focusing on one particular review related to miRNAs in Alzheimer disease (10). We finally describe a strategy which is not biased by the current knowledge and we argue it should be applied in preference to future studies based on a similar design.

## MATERIALS AND METHODS

### Identification of miRNA targets

Three resources were used to identify miRNA targets. mirTarBase (11) (version 4.5) is a database of experimentally validated miRNA-target interactions. For human, 1324 targets are associated to 344 miRNAs. TargetScan (12–14) is an online software provided by MIT for prediction of miRNA targets. For human, 11 161 targets are predicted for 277 miRNAs. The Thomson-Reuters MetaBase (http://thomsonreuters.com/metabase/) is a comprehensive manually curated database of mammalian biology and medicinal chemistry data. For human, 2247 targets are associated to 699 miRNAs.

### Pathways

Two pathway databases were used in the frame of this study. The KEGG.db package (15) provides 229 KEGG (16) pathways. The Thomson-Reuters MetaBase provides a list of 1283 pathways.

### Enrichment analyses

All enrichment analyses described in this study are based on the hypergeometric test:

\begin{equation*} \begin{array}{*{20}l} {P{\rm - }value = } {\frac{{R!n!(N - R)!(N - n)!}}{{N!}}\sum\limits_{i = r}^{\min (n,R)} {\frac{1}{{i!(R - i)!(n - i)!(N - R - n + i)i}}} } \end{array} \end{equation*}

with *N* the number of elements in the universe under focus, *R* the number of element in the query, *n* the number of elements in the reference and *r* the overlap between the query and the reference. Correction for multiple testing was done using Benjamini–Hochberg method (17) and significantly enriched pathways were selected according to a false discovery rate (FDR) <0.05.

## RESULTS

In order to compare the different strategies for associating pathways to miRNA signatures, the following results are mainly derived from a re-analysis of miRNAs differentially expressed in Alzheimer's disease (AD) (10), one of our research interests. In his review, Satoh identified 16 miRNAs over-expressed (AD-up) and 113 miRNAs under-expressed (AD-down) in AD patients compared to healthy controls.

All of the following analyses were based on considering pathways as lists of protein coding genes. Thus, one important step common to all the strategies is to first identify the target genes of miRNAs. Several resources are available to perform this step (see ‘Materials and Methods’ section). Again, in order to compare the strategies as such, we deliberately focused on one of these resources: mirTarBase (11). AD-up and AD-down miRNAs were compared to miRNA identifiers available in mirTarBase leading to slightly smaller lists of 16 and 99 miRNAs (Supplementary Table S1). Also the main list of pathways with which the following analyses were performed are from the KEGG database (16) as provided by the KEGG.db package (15).

### Strategy 1: indirect enrichment of miRNA target genes in native pathways

**Figure 1.**
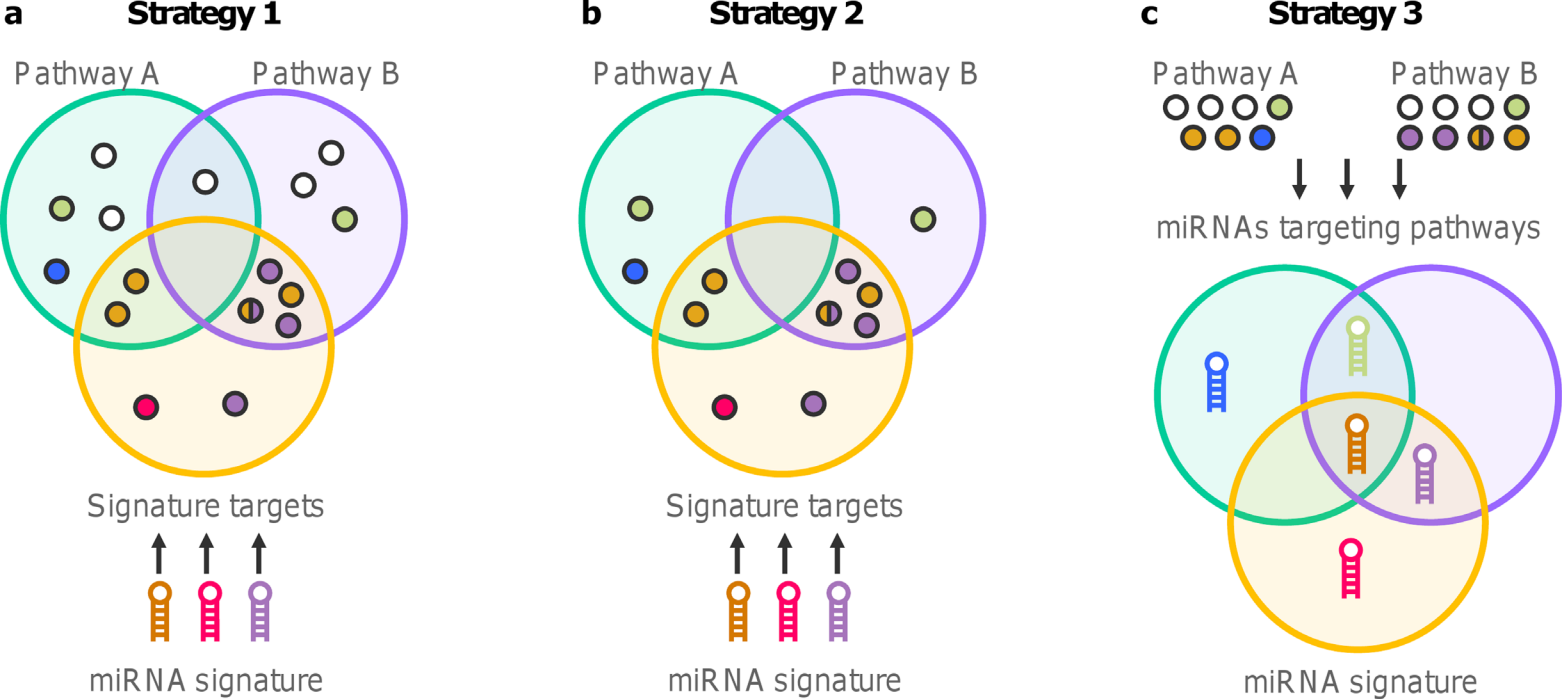
Strategies to identify pathways associated to a miRNA signature. Circles represent protein coding genes and hairpins miRNAs. Gene having the same color of a miRNA are targeted by this miRNA. White genes are not known to be targeted by any miRNA. (**a**) Strategy 1: targets of the miRNAs of interest are identified using *in silico* resources and then compared to protein coding genes belonging to each native pathway. (**b**) Strategy 2: same as strategy 1 but pathways are tailored to only keep genes targeted by at least one miRNA. (**c**) Strategy 3: pathways of protein coding genes are converted in lists of miRNAs that target at least one of their genes. Then the miRNA signature is directly compared to miRNAs-converted pathways.

The most straightforward and widely used strategy to identify pathways associated to a list of miRNAs is to perform an enrichment analysis of the miRNA target genes (Figure 1a) (e.g. (3,4,6–10)). First, genes targeted by any miRNA of interest are identified using a reference database or a prediction algorithm. Then the significance of the overlap between target genes and pathway genes is measured by an enrichment analysis (see ‘Materials and Methods’). This strategy was applied with the AD-up and AD-down lists of miRNAs.

According to mirTarBase, 70 genes are targeted by at least one of the 16 AD-up miRNAs. These target genes are significantly enriched (FDR < 0.05) in genes of 38 KEGG pathways (Supplementary Table S1—AD-up S1 pathways). Similarly, the 99 AD-down miRNAs led to the identification of 762 target genes (Supplementary Table S1—AD-down targets) significantly enriched in genes of 73 KEGG pathways (Supplementary Table S1—AD-down S1 pathways). Intriguingly, 37 pathways are common to the two lists of miRNAs even if, by definition, no miRNA belongs to both lists. This observation raised doubts about the validity of the approach and the results it generates.

In order to assess the specificity of this strategy, the same workflow was applied to 1000 random selections of 16 (RAND-16) and 99 (RAND-99) miRNAs, lists of lengths equal to the AD-up and AD-down miRNAs respectively. On average 120 genes (SD = 46) are targeted by RAND-16 and 555 (SD = 78) by RAND-99 miRNAs. Among the 229 KEGG pathways, 14 are enriched (FDR < 0.05) in at least 90% of RAND-16 targets and 64 KEGG pathways are enriched in at least 10% of RAND-16 targets. These 64 pathways include 37 of the previously identified 38 AD-up S1 pathways (97%). Similarly, 51 KEGG pathways are enriched in at least 90% of RAND-99 targets and 78 pathways are enriched in at least 10% of RAND-99 targets, including 72 of the 73 AD-down S1 pathways (99%). This result shows that this broadly used strategy in miRNA-related literature is biased and leads to highly unspecific outcomes, i.e. the same pathways are typically identified whatever the initial input list of miRNAs.

**Figure 2.**
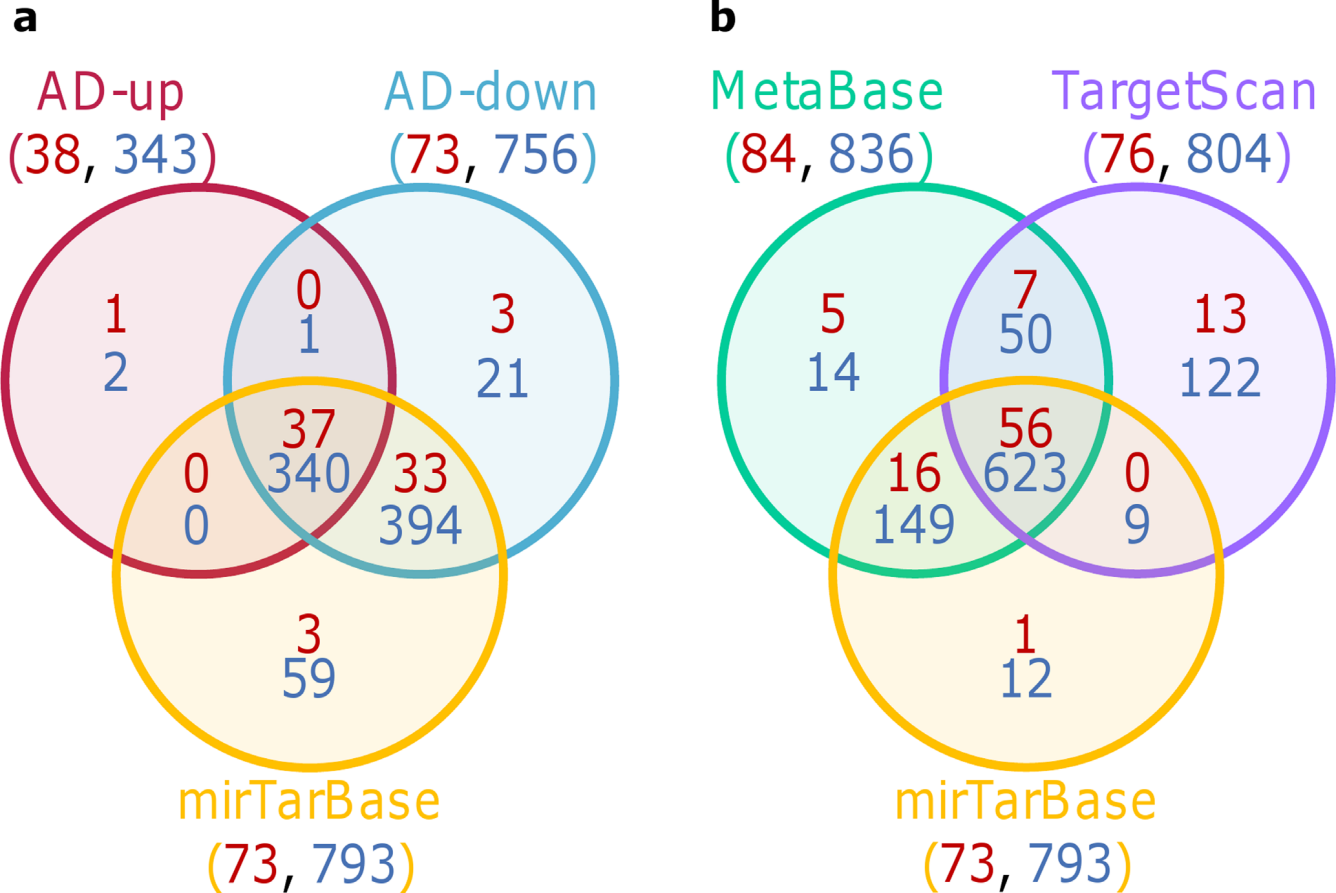
Pathways associated to miRNA signatures when applying strategy 1. (**a**) AD-up S1 pathways, AD-down S1 pathways and pathways enriched in genes targeted by at least one miRNA according to mirTarBase. (**b**) Pathways enriched in genes targeted by at least one miRNA according to MetaBase, TargetScan or mirTarBase. Numbers in red correspond to KEGG pathways and those in blue to MetaBase pathways.

The results with random selections of mirRNAs indicate that the whole list of miRNA targets is biased for a subset of biological functions. In order to test this hypothesis, an enrichment analysis was performed starting from the whole list of 1324 genes targeted by at least one miRNA in mirTarBase. 73 KEGG pathways are significantly enriched (FDR < 0.05) with these 1324 miRNA target genes and they mainly relate to cancer and cell cycle (Supplementary Table S2). Table 1 shows the 20 most significant ones. When compared to previous AD use case, it turned out that 37 out of 38 AD-up and 70 out of 73 AD-down S1 pathways are common to pathways non-specifically enriched in whole list of miRNA target genes (Figure 2a). This result shows that the targets available in mirTarBase are strongly biased for some KEGG pathways.

**Table 1.** Top 20 pathways enriched in all protein coding genes targeted by at least one miRNA according to mirTarBase

	Rank	*P*-value	FDR	Alzheimer's disease (10)	Chordoma (3)	Developing human brain (18)	Peripheral arterial disease (19)
Pathways in cancer	1	2.68E-61	6.15E-59	YES	YES		YES
Prostate cancer	2	3.17E-32	3.63E-30	YES	YES	YES	YES
Pancreatic cancer	3	2.5E-29	1.72E-27	YES	YES	YES	YES
Chronic myeloid leukemia	3	3.01E-29	1.72E-27	YES		YES	YES
Colorectal cancer	5	1.09E-26	4.99E-25	YES			YES
Focal adhesion	6	1.5E-23	5.71E-22	YES	YES		YES
Neurotrophin signaling pathway	7	3.65E-23	1.19E-21		YES		YES
MAPK signaling pathway	8	7.01E-22	2.01E-20	YES	YES		YES
Small cell lung cancer	9	8.74E-22	2.23E-20	YES	YES		YES
Osteoclast differentiation	10	2.81E-21	5.84E-20				YES
Cell cycle	10	2.81E-21	5.84E-20	YES	YES		YES
Bladder cancer	12	7.62E-21	1.45E-19	YES			YES
Renal cell carcinoma	13	2.96E-20	5.21E-19	YES	YES	YES	YES
Chagas disease (American trypanosomiasis)	14	1E-19	1.64E-18				YES
Toxoplasmosis	15	5.59E-18	8.24E-17				YES
Melanoma	15	5.76E-18	8.24E-17	YES			YES
Endometrial cancer	17	8.94E-18	1.2E-16	YES			YES
Toll-like receptor signaling pathway	18	1.23E-17	1.57E-16				YES
T cell receptor signaling pathway	19	3.15E-17	3.8E-16		YES	YES	YES
Glioma	20	9.45E-17	1.08E-15	YES	YES	YES	YES

The first three columns show the significance of the association. The remaining column indicate pathways identified in different studies focused on different topics.

In order to check if the bias is only related to mirTarBase and KEGG pathways, the same approach was applied using other resources for miRNA target identification and biological pathways. Many KEGG pathways are also enriched with two other lists of miRNA targets provided by targetScan (12–14) and by the Thomson-Reuters MetaBase (Figure 2b). Moreover, a similar bias was observed when performing the enrichment analysis on the MetaBase pathways whatever the source of miRNA targets (Figure 2a and b). Taken together these results show that the bias is common to different methods to identify miRNA targets or to different biological pathways resources.

In his review, Satoh (10) describes 20 top KEGG pathways related to miRNAs down regulated in AD brains (Supplementary Table S2). However, according to our results, these pathways strongly overlap with the non-specifically identified ones. Thus no conclusion can be drawn about the involvement of the miRNAs under expressed in AD brain in the regulation of these 20 pathways, contrary to what the author claims.

Other similar studies related to miRNA signatures use this strategy to identify impacted pathways. For instance one identified miRNAs differentially expressed during human brain development (18), another those deregulated in peripheral arterial disease (19) and a third one in chordomas (3). Most of the pathways described in these publications are also enriched with the whole list of miRNA targets (respectively 9/11, 51/56 and 34/44), preventing any conclusion about their specific regulation by miRNAs associated to the conditions under focus (Table 1 and Supplementary Table S2). These results show a strong negative impact of this biased strategy in miRNA-related literature and bring out the need to establish a new unbiased analysis strategy for pathway identification from miRNA signatures.

Many pathways identified by the approach described above, whatever the databases used, are related to cancer which suggests a knowledge bias in this field for microRNA compared to protein coding genes. To test this hypothesis, the relative representations of miRNA and protein coding genes in cancer-related scientific literature were compared using text-mining approach (Supplementary Information). The analysis confirms a significant bias in current knowledge related to the role of miRNA in diseases toward neoplastic and cancer-related processes. When performing analysis, such as pathway identification in this case, the method used should not be influenced by any bias in the knowledge representation.

### Strategy 2: indirect enrichment of miRNA target genes in tailored pathways

In the first strategy, all the genes belonging to each pathway were taken into account to compute the significance of the overlap with the list of targets of the miRNA signature. However, many of these genes are not known to be targeted by a miRNA and we previously showed that miRNA targets are significantly enriched with genes from some pathways, mainly related to cancer. Thus, these pathways have more chances to be selected if this imbalance is not taken into account. The most straightforward way to handle this bias is to tailor pathways to only keep genes targeted by at least one miRNA, using the same resource to identify targets of the list of miRNAs of interest (Figure 1b). This new strategy was again applied to AD-up and AD-down lists of miRNAs.

When applying this strategy, 5 KEGG pathways (AD-up S2 pathways) are enriched in AD-up targets (FDR < 0.05) and 3 KEGG pathways (AD-down S2 pathways) show enrichment for AD-down targets. To assess the specificity of this strategy, the same workflow was applied on RAND-16 and RAND-99 miRNAs. 12 KEGG pathways are enriched in at least 10% of RAND-16 targets, including all the 5 AD-up S2 pathways. Similarly, 18 KEGG pathways are enriched in at least 10% of RAND-99 targets, including all the three AD-down S2 pathways (Supplementary Table S1). These results show that this alternative strategy, based on the filtering of pathway resources for miRNA target genes, is also not specific even if fewer pathways are finally selected compared to the first strategy.

The low specificity of this approach could be related to the identification of genes targeted by miRNAs of interest. Indeed, since the genes on which the enrichment analysis is performed are selected indirectly, the number of miRNAs targeting each gene may affect the probability to include them in the enrichment analysis, with genes targeted by many miRNAs having more chances to be selected in the first step of the strategy. To test this hypothesis an enrichment analysis was performed on the whole list of targets in mirTarBase but also on the 1000, 500, 100 and 50 most commonly targeted genes. By design, no pathway is significantly enriched with the whole list of 1324 miRNA target genes and only one is enriched with the top 1000 most targeted genes. In contrast, 20, 12 and 18 KEGG pathways were significantly enriched (FDR < 0.05) respectively with the top 500, 100 and 50 targeted genes, most of these pathways being related to cancer biology (Supplementary Table S2). This result shows that genes having more chances to be selected in the first step of this approach are already significantly enriched in some pathways, thereby unbalancing the second step of the strategy.

**Figure 3.**
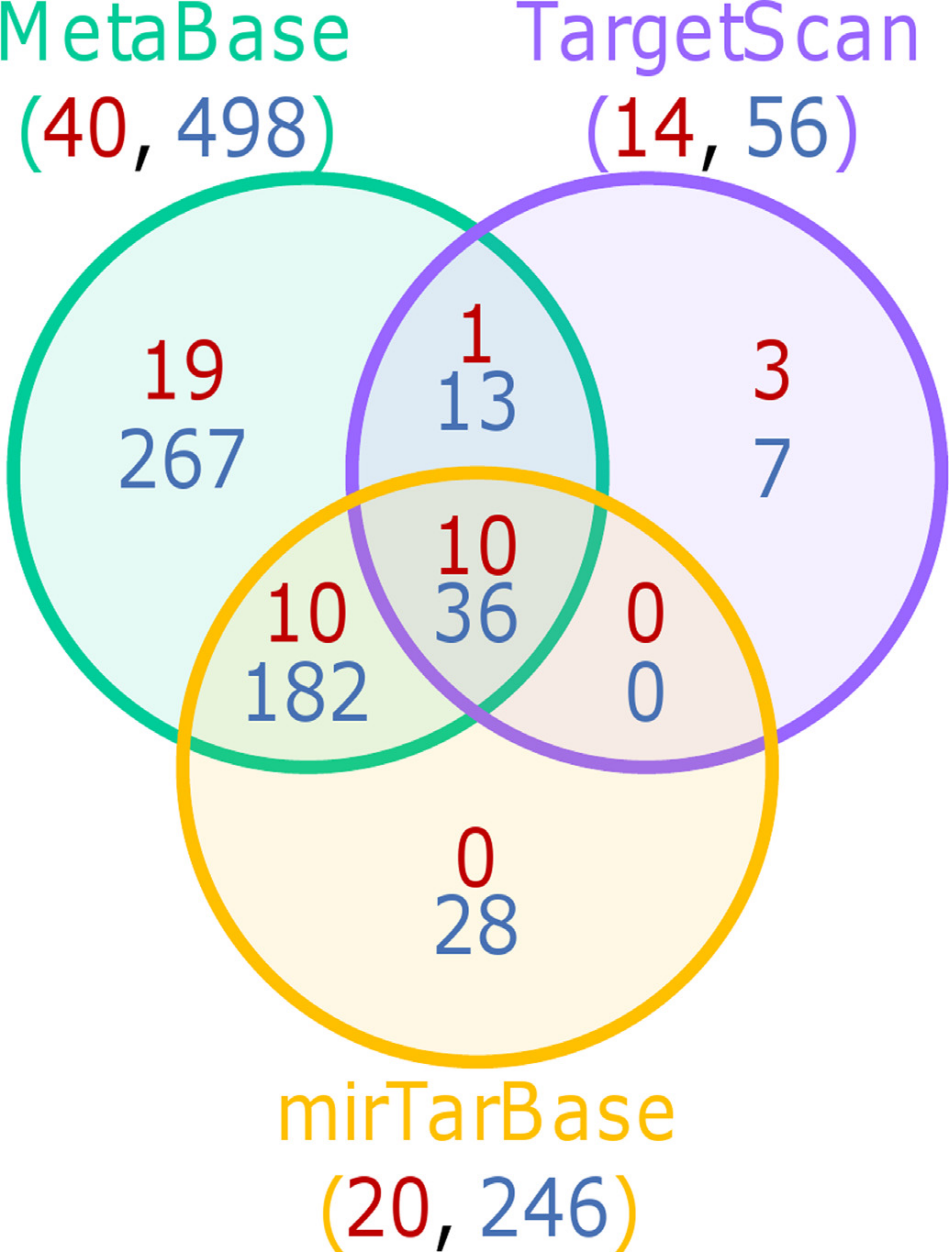
Pathways enriched in the top 500 genes targeted by miRNAs according to MetaBase, TargetScan or mirTarBase when applying strategy 2. Numbers in red correspond to KEGG pathways and those in blue to MetaBase pathways.

Again, to check the dependence of this result toward the tools and databases, the same workflow was applied using alternative resources. The same kind of bias is observed when using other sources for miRNA target identification and when performing the enrichment analysis on the MetaBase pathways (Figure 3). This result indicates that this bias is related to the method as such and not to the knowledge resources it uses.

### Strategy 3: direct enrichment of miRNAs in converted pathways

In order to avoid bias related to the previously described indirect identification of the genes to be included in the enrichment analysis, a third strategy was set-up (Figure 1c). First, pathways of protein coding genes are converted in lists of miRNAs that target at least one of these genes. Then the enrichment analysis is conducted by direct comparison of the list of miRNAs of interest to the lists of miRNAs previously associated to the different pathways. This strategy ensures that a miRNA is only represented once in a pathway whatever the number of its target genes in this particular pathway.

After applying this strategy to the AD use case, not a single pathway was found to be significantly enriched in the 16 AD-up miRNAs whereas 81 pathways were significantly enriched (FDR < 0.05) in the 99 AD-down miRNAs (AD-down S3 pathways) (Supplementary Table S1). Again, to assess whether this new strategy leads to an improvement in specificity, this methodology was applied to 1000 random selections of 16 or 99 miRNAs. No single pathway was selected more than three times on the 1000 trials (FDR < 0.05), clearly supporting the specificity of this approach.

**Figure 4.**
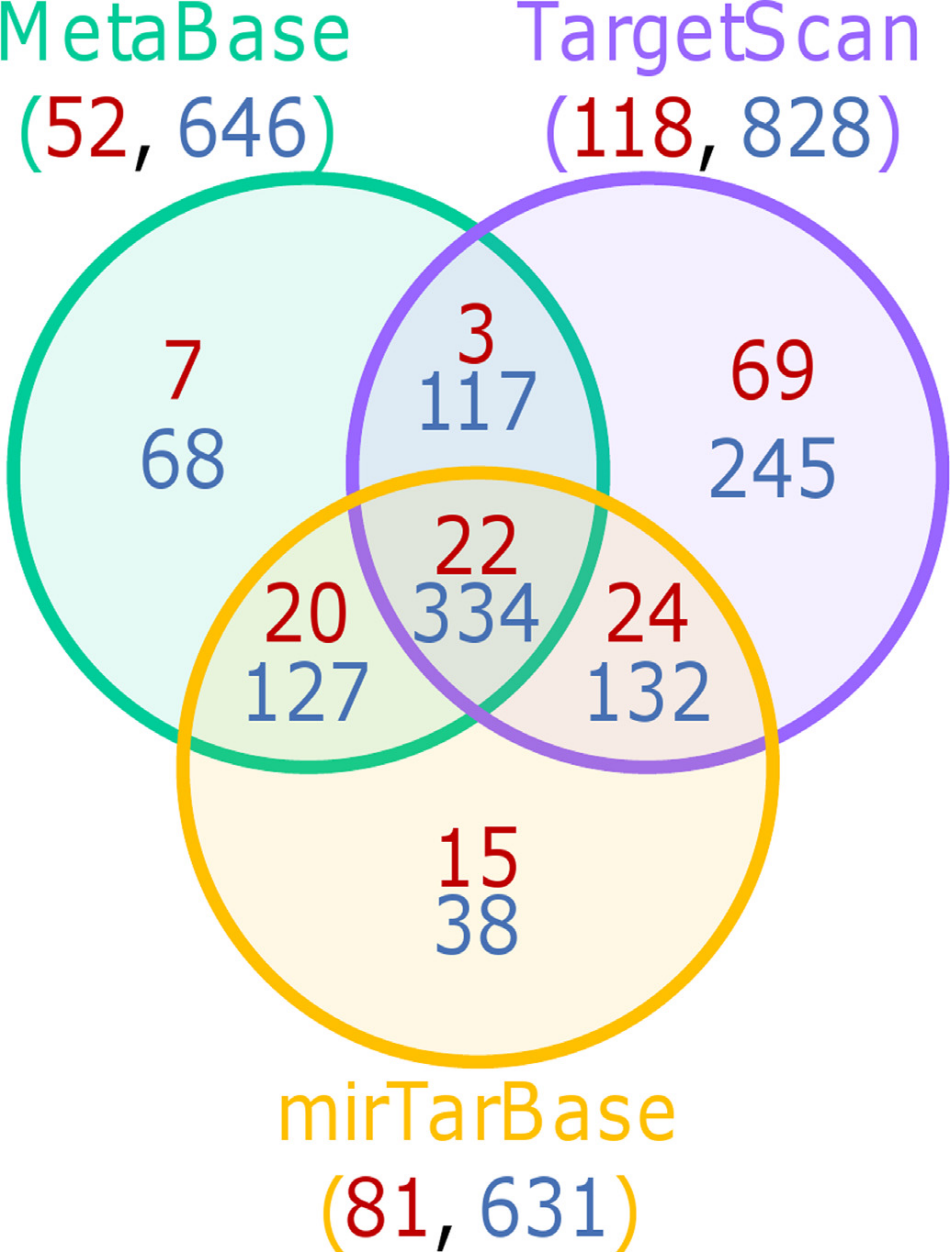
Pathways associated to AD-down miRNAs when applying strategy 3. miRNAs targeting protein coding genes in the different pathways were identified using either MetaBase, TargetScan or mirTarBase. Numbers in red correspond to KEGG pathways and those in blue to MetaBase pathways.

Again, different tools to identify miRNA targets were compared in order to assess their impact on pathways associated to the miRNA signature. Beside a strong overlap between the different tools, a significant number of pathways are identified only when using only one of them (Figure 4). Also, the same strategy was applied using MetaBase pathways and lead to the identification of 631 pathways enriched in AD-down miRNAs and no pathway enriched in AD-up miRNAs. Again, starting from 1000 random selections of 16 or 99 miRNAs, no Metabase pathway was selected more than twice (FDR < 0.05). This observation reflects a different representation of the knowledge in the tools and resources used and, more importantly, that the new method can be applied to different resources, their knowledge bias not affecting the statistical relevance of the final results.

Nevertheless, the number of pathways significantly enriched with AD-down miRNAs is surprisingly large (81 KEGG and 631 Metabase pathways) considering the number of miRNAs (99). This result indicates that these pathways share many miRNAs.

**Figure 5.**
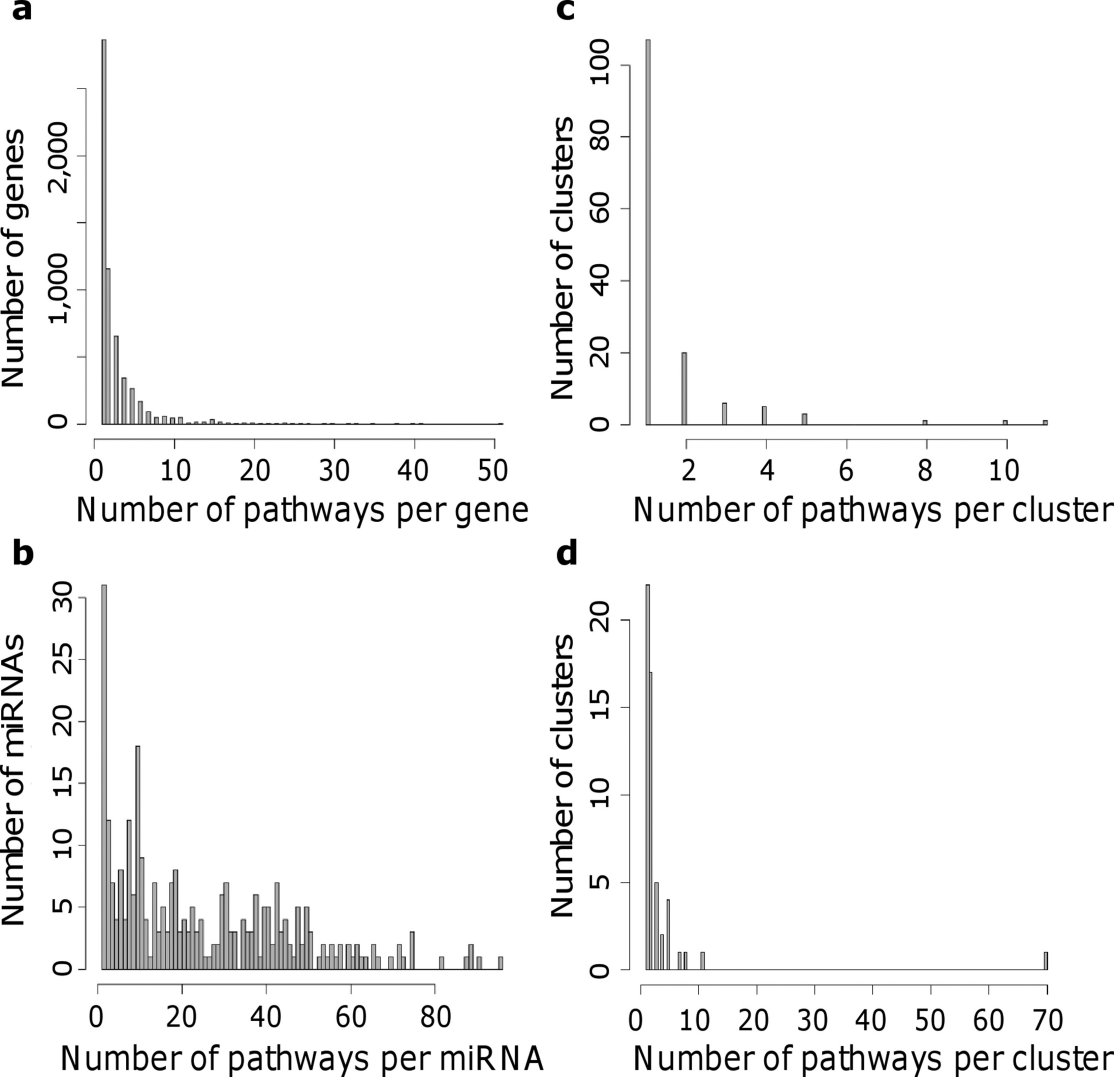
KEGG pathway redundancy at the levels of protein coding genes and miRNAs. (**a**) Distribution of the number of pathways associated to each Entrez gene ID in the KEGG.db package. (**b**) Distribution of the number of pathways associated to each miRNA using mirTarBase information. (**c**) Distribution of the number of pathways per cluster of pathways sharing on average at least 20% of Entrez gene ID. (**d**) Distribution of the number of pathways per cluster of pathways sharing on average at least 20% of associated miRNAs using mirTarBase information.

To test this hypothesis, the number of KEGG pathways associated to each miRNA was compared to the number of pathways containing each original protein coding gene. As shown in Figure 5, there are many miRNAs associated to more than 10 pathways (Figure 5b) whereas most of the protein coding genes are associated to <5 (Figure 5a). This result shows that pathway information is much more redundant at the miRNA level than at the gene level.

The structure of this relative overlap was also compared applying a hierarchical clustering on the Jaccard index computed for each pathway pair at the level of protein coding genes ($J(A,B) = \frac{{|A \cap B|}}{{|A \cup B|}}$) (Supplementary Figure S1) and at the level of miRNAs (Supplementary Figure S2). Clusters of pathways were defined according to an average Jaccard index of 20%. The number of pathways per cluster is shown in Figure 5c and d. On one hand, most of the pathways do not share more than 20% of their protein coding genes with any other pathway (Figure 5c). On the other hand, there is a large cluster of 70 pathways sharing on average more than 20% of their associated miRNAs and many other pathways are related to each other at this level (Figure 5d).

This result indicates that pathway enrichments with genes are generally independent of each other whereas it's not the case with miRNAs. Indeed, the majority (58) of the 81 pathways significantly enriched in the 99 miRNAs under expressed in AD brains belong to the large cluster of 70 pathways (Supplementary Table S1). It is therefore difficult to identify which of those are relevant to the disease.

Nevertheless, 11 pathways which are significantly enriched in the 99 AD-down miRNAs belong to small clusters (≤2 pathways). These 11 pathways are more probably specific to the study under focus: miRNA regulations involved in Alzheimer's disease. Strengthening this hypothesis, the Alzheimer's disease KEGG pathway belongs to this list of 11 pathways. The FDR of the overlap between this pathway and the 99 AD-down miRNAs is 0.007. This pathway was not identified by the two previous strategies.

The same strategy was applied on various additional lists of miRNA identified by former studies as being differentially expressed during the development of human brain (18), in peripheral arterial disease (19) or in chordomas (3). 22 KEGG pathways were found to be significantly associated to miRNA over expressed in chordomas (FDR < 0.05) that also differ from the ones identified in original publication (Supplementary Table S3). Not a single pathway was found to be significantly enriched in the other miRNA lists, making their interpretation difficult from a pathway point of view.

## DISCUSSION

Here we show that the most commonly applied *in silico* strategy to infer pathways that are under the regulation of a miRNA signature is not specific and leads to the systematic identification of highly related biological processes, namely cell cycle and cancer biology. In this indirect approach, targets of the miRNAs are compared to pathways of protein coding genes. Many published results related to the functional interpretation of differentially expressed miRNAs are based on this biased indirect analysis (e.g. (3,10,18,19)) and identify similar biological processes even if the conditions of interest are very different. Our results show that, the conclusions of such studies regarding inferred regulated pathways cannot be trusted and should be reviewed after new unbiased analyses. Moreover, these results also indicate that tools applying this strategy, such as DIANA mirPath (20), should be modified in order to take this bias into account.

Two alternatives are also explored to avoid this bias. One is highly related to the previous strategy with the exception that protein coding genes taken into account in the pathways are limited to known targets of miRNAs. We showed that this other indirect strategy is also biased because of the knowledge about miRNA biology which is highly related to cancer. As recently discussed by Mørk (21), this bias could be related to a study imbalance or to the underlying function of miRNAs as such. In any case, this has to be taken into account when performing analyses using the current knowledge, such as pathway analyses.

The last strategy we explored is the reverse of the first two. Pathways of protein coding genes are converted into lists of miRNAs targeting at least one gene of the original pathway, avoiding overcounting the miRNAs targeting multiple genes belonging to the same pathway. Then, the enrichment analysis is directly performed with the miRNA signature under focus. Recently, we applied this approach to miRNA signatures of various mouse models of epilepsy (22) and we proposed a role for these miRNAs in the regulation of inflammatory pathways in this pathology. This approach is not biased by the current knowledge and results are specific to each miRNA signature. However, we also show that many pathways share a significant number of miRNAs often leading to their co-identification. In order to avoid this issue and also to decrease useless multiple testing, these similar converted pathways could be aggregated before any enrichment analysis.

More generally, enrichment in protein coding gene pathways should be performed with care when not directly starting from genes but, for example, from their upstream regulators such as miRNAs, transcription factors or protein kinases. In such case, the alternative strategy established in the frame of this study could be beneficially applied. In any case, appropriate negative controls should always be included in the analysis in order to assess any potential bias related to the chosen strategy.

## SUPPLEMENTARY DATA

Supplementary Data are available at NAR Online.

SUPPLEMENTARY DATA
