# Supplementary material for: Pathway analysis from lists of microRNAs: common pitfalls and alternative strategy
Source: Nucleic Acids Res. 2015 Mar 23;43(7):3490–7. doi: 10.1093/nar/gkv249 (PMC4402548; doi:10.1093/nar/gkv249)
Supplement: SUPPLEMENTARY DATA [file supp_43_7_3490__index.html]

Pathway analysis from lists of microRNAs: common pitfalls and alternative strategy — Pathway analysis from lists of microRNAs: common pitfalls and alternative strategy — SUPPLEMENTARY DATA 

# Pathway analysis from lists of microRNAs: common pitfalls and alternative strategy

## SUPPLEMENTARY DATA

**Files in this Data Supplement:**

- SUPPLEMENTARY DATA
- SUPPLEMENTARY DATA
- SUPPLEMENTARY DATA
- SUPPLEMENTARY DATA
