# Supplementary material for: Pathway analysis from lists of microRNAs: common pitfalls and alternative strategy
Source: Nucleic Acids Res. 2015 Mar 23;43(7):3490–7. doi: 10.1093/nar/gkv249 (PMC4402548; doi:10.1093/nar/gkv249)
Supplement: SUPPLEMENTARY DATA [file supp_gkv249_nar-03642-n-2014-File008.pdf]

Supplementary figures and supplementary table legends

Pathway analysis from lists of microRNAs : common pitfalls and alternative strategy

Patrice Godard and Jonathan van Eyll

Supplementary information

The microRNA knowledge is biased for cancer biology

Many pathways identified by the strategy 1 are related to cancer which indicates a knowledge bias in this field for microRNA compared to protein coding genes.

To test this hypothesis, the relative representations of miRNA genes and protein coding genes in cancer-related scientific literature were compared using the SCAIview test mining tool(1). This tool allows a quantitative analysis of ontology-mapped terms co-occurence in the entire collection of Pubmed abstracts and extracted all Mesh diseases co-cited with either a miRNA or a protein coding genes.

Using an hypergeometric test, 1093 Mesh diseases were identified as significantly enriched with miRNA and 3320 with protein coding genes. Among them 17.5% (163) and 8.5% (259) respectively are neoplastic diseases. The difference between the proportions of neoplastic diseases associated to miRNA and protein coding genes is highly significant according to a Fisher test (p-value < 10^{-10}). This result shows a significant bias in current the knowledge related to the role of miRNA in diseases towards neoplastic and cancer-related processes. As recently discussed by Mørk(2), this bias could be related to a study imbalance or to the underlying function of miRNA as such. Anyway it has to be taken into account when performing analyses using the current knowledge, such as pathways analyses.

Method for literature analysis

The list of Mesh diseases co-cited with either protein coding genes or miRNA genes was obtained using SCAIview Pharma 1.0 running on the collection of Pubmed abstracts mentioning a Mesh disease as available on 19 November 2013 (13,145,693 abstracts). Mesh diseases significantly co-cited (FDR < 0.001) with either a protein coding gene or a miRNA were obtained applying an hypergeometric test.

To differentiate neoplastic Mesh diseases from other Mesh diseases, the Mesh disease ontology(3) was downloaded from Mesh website (http://www.nlm.nih.gov/mesh/2014/download/mtr_smp.txt) and all diseases falling under the node “C04 – Neoplasms” were classified as neoplastic compared to others.

Supplementary Figure 1

Hierarchical clustering on the Jaccard index computed for each pathway couple at the level of protein coding gene. The average linkage method was used to build the tree. Cluster of pathways sharing on average at least 20% of Entrez gene ID according to the KEGG.db(4) package are highlighted.

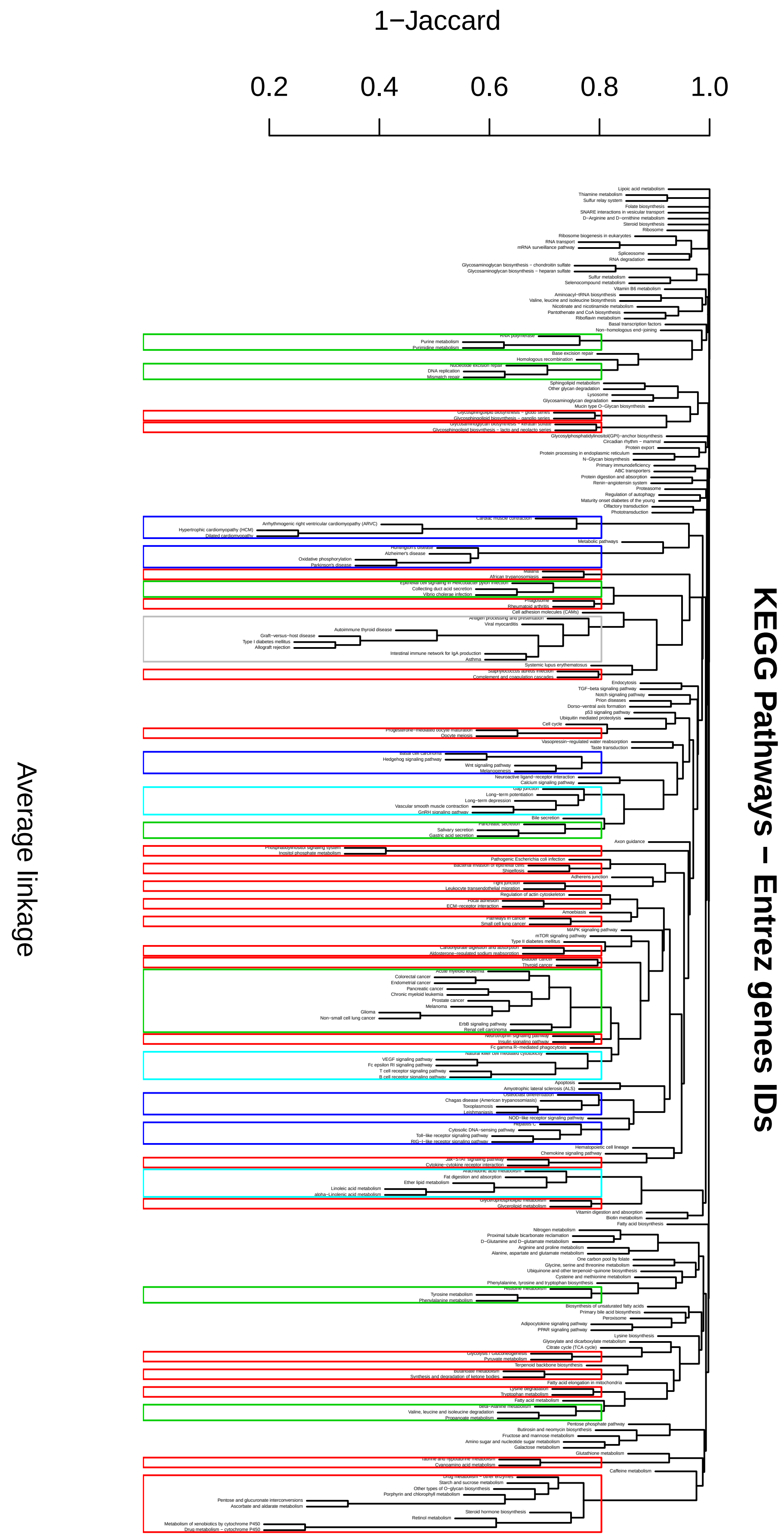

1−Jaccard
0.2
0.4
0.6
0.8
1.0
KEGG Pathways – Entrez genes IDs
Average linkage
Lipoic acid metabolism
Thiamine metabolism
Sulfur relay system
Folate biosynthesis
SNARE interactions in vesicular transport
D−Arginine and D−ornithine metabolism
Steroid biosynthesis
Ribosome
Ribosome biogenesis in eukaryotes
RNA transport
mRNA surveillance pathway
Spliceosome
RNA degradation
Glycosaminoglycan biosynthesis − chondroitin sulfate
Glycosaminoglycan biosynthesis − heparan sulfate
Sulfur metabolism
Selenocompound metabolism
Vitamin B6 metabolism
Aminoacyl−tRNA biosynthesis
Valine, leucine and isoleucine biosynthesis
Nicotinate and nicotinamide metabolism
Pantothenate and CoA biosynthesis
Riboflavin metabolism
Basal transcription factors
Non−homologous end−joining
RNA polymerase
Purine metabolism
Pyrimidine metabolism
Base excision repair
Homologous recombination
Nucleotide excision repair
DNA replication
Mismatch repair
Sphingolipid metabolism
Other glycan degradation
Lysosome
Glycosaminoglycan degradation
Mucin type O−Glycan biosynthesis
Glycosphingolipid biosynthesis − globo series
Glycosphingolipid biosynthesis − ganglio series
Glycosaminoglycan biosynthesis − keratan sulfate
Glycosphingolipid biosynthesis − lacto and neolacto series
Glycosylphosphatidylinositol(GPI)−anchor biosynthesis
Circadian rhythm − mammal
Protein export
Protein processing in endoplasmic reticulum
N−Glycan biosynthesis
Primary immunodeficiency
ABC transporters
Protein digestion and absorption
Renin−angiotensin system
Proteasome
Regulation of autophagy
Maturity onset diabetes of the young
Olfactory transduction
Phototransduction
Cardiac muscle contraction
Arrhythmogenic right ventricular cardiomyopathy (ARVC)
Hypertrophic cardiomyopathy (HCM)
Dilated cardiomyopathy
Metabolic pathways
Huntington's disease
Alzheimer's disease
Oxidative phosphorylation
Parkinson's disease
Malaria
African trypanosomiasis
Epithelial cell signaling in Helicobacter pylori infection
Collecting duct acid secretion
Vibrio cholerae infection
Phagosome
Rheumatoid arthritis
Cell adhesion molecules (CAMs)
Antigen processing and presentation
Viral myocarditis
Autoimmune thyroid disease
Graft−versus−host disease
Type I diabetes mellitus
Allograft rejection
Intestinal immune network for IgA production
Asthma
Systemic lupus erythematosus
Staphylococcus aureus infection
Complement and coagulation cascades
Endocytosis
TGF−beta signaling pathway
Notch signaling pathway
Prion diseases
Dorso−ventral axis formation
p53 signaling pathway
Ubiquitin mediated proteolysis
Cell cycle
Progesterone−mediated oocyte maturation
Oocyte meiosis
Vasopressin−regulated water reabsorption
Taste transduction
Basal cell carcinoma
Hedgehog signaling pathway
Wnt signaling pathway
Melanogenesis
Neuroactive ligand−receptor interaction
Calcium signaling pathway
Gap junction
Long−term potentiation
Long−term depression
Vascular smooth muscle contraction
GnRH signaling pathway
Bile secretion
Pancreatic secretion
Salivary secretion
Gastric acid secretion
Axon guidance
Phosphatidylinositol signaling system
Inositol phosphate metabolism
Pathogenic Escherichia coli infection
Bacterial invasion of epithelial cells
Shigellosis
Adherens junction
Tight junction
Leukocyte transendothelial migration
Regulation of actin cytoskeleton
Focal adhesion
ECM−receptor interaction
Amoebiasis
Pathways in cancer
Small cell lung cancer
MAPK signaling pathway
mTOR signaling pathway
Type II diabetes mellitus
Carbohydrate digestion and absorption
Aldosterone−regulated sodium reabsorption
Bladder cancer
Thyroid cancer
Acute myeloid leukemia
Colorectal cancer
Endometrial cancer
Pancreatic cancer
Chronic myeloid leukemia
Prostate cancer
Melanoma
Glioma
Non−small cell lung cancer
ErbB signaling pathway
Renal cell carcinoma
Neurotrophin signaling pathway
Insulin signaling pathway
Fc gamma R−mediated phagocytosis
Natural killer cell mediated cytotoxicity
VEGF signaling pathway
Fc epsilon RI signaling pathway
T cell receptor signaling pathway
B cell receptor signaling pathway
Apoptosis
Amyotrophic lateral sclerosis (ALS)
Osteoclast differentiation
Chagas disease (American trypanosomiasis)
Toxoplasmosis
Leishmaniasis
NOD−like receptor signaling pathway
Hepatitis C
Cytosolic DNA−sensing pathway
Toll−like receptor signaling pathway
RIG−I−like receptor signaling pathway
Hematopoietic cell lineage
Chemokine signaling pathway
Jak−STAT signaling pathway
Cytokine−cytokine receptor interaction
Arachidonic acid metabolism
Fat digestion and absorption
Ether lipid metabolism
Linoleic acid metabolism
alpha−Linolenic acid metabolism
Glycerophospholipid metabolism
Glycerolipid metabolism
Vitamin digestion and absorption
Biotin metabolism
Fatty acid biosynthesis
Nitrogen metabolism
Proximal tubule bicarbonate reclamation
D−Glutamine and D−glutamate metabolism
Arginine and proline metabolism
Alanine, aspartate and glutamate metabolism
One carbon pool by folate
Glycine, serine and threonine metabolism
Ubiquinone and other terpenoid−quinone biosynthesis
Cysteine and methionine metabolism
Phenylalanine, tyrosine and tryptophan biosynthesis
Histidine metabolism
Tyrosine metabolism
Phenylalanine metabolism
Biosynthesis of unsaturated fatty acids
Primary bile acid biosynthesis
Peroxisome
Adipocytokine signaling pathway
PPAR signaling pathway
Lysine biosynthesis
Glyoxylate and dicarboxylate metabolism
Citrate cycle (TCA cycle)
Glycolysis / Gluconeogenesis
Pyruvate metabolism
Terpenoid backbone biosynthesis
Butanoate metabolism
Synthesis and degradation of ketone bodies
Fatty acid elongation in mitochondria
Lysine degradation
Tryptophan metabolism
Fatty acid metabolism
beta−Alanine metabolism
Valine, leucine and isoleucine degradation
Propanoate metabolism
Pentose phosphate pathway
Butirosin and neomycin biosynthesis
Fructose and mannose metabolism
Amino sugar and nucleotide sugar metabolism
Galactose metabolism
Glutathione metabolism
Taurine and hypotaurine metabolism
Cyanoamino acid metabolism
Caffeine metabolism
Drug metabolism − other enzymes
Starch and sucrose metabolism
Other types of O−glycan biosynthesis
Porphyrin and chlorophyll metabolism
Pentose and glucuronate interconversions
Ascorbate and aldarate metabolism
Steroid hormone biosynthesis
Retinol metabolism
Metabolism of xenobiotics by cytochrome P450
Drug metabolism − cytochrome P450

Supplementary Figure 2

Hierarchical clustering on the Jaccard index computed for each pathway couple at the level of microRNA. The average linkage method was used to build the tree. Cluster of pathways sharing on average at least 20% of associated miRNAs using mirTarBase(5) information are highlighted.

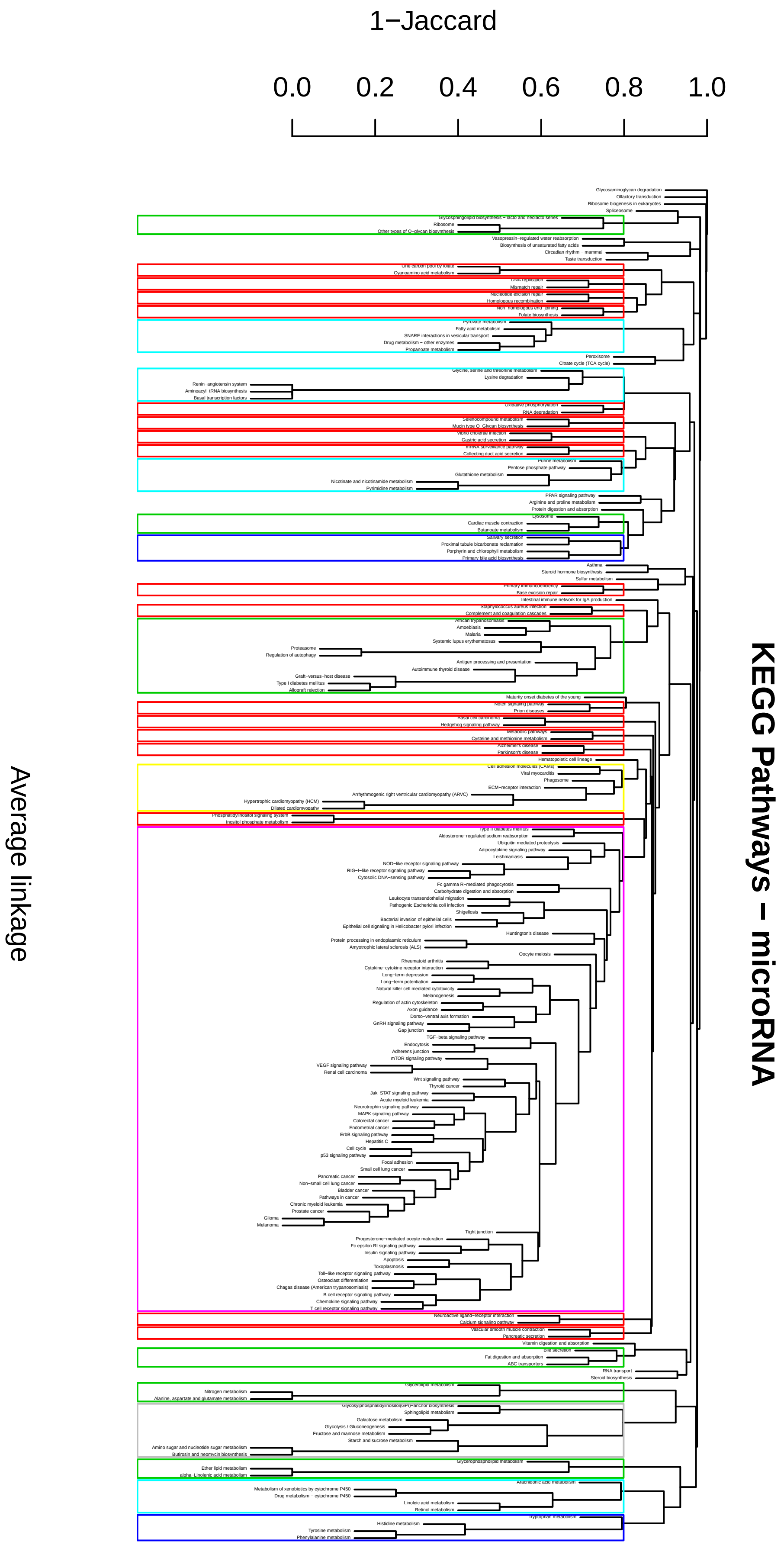
1−Jaccard
0.0
0.2
0.4
0.6
0.8
1.0
Average linkage
KEGG Pathways – microRNA
Glycosaminoglycan degradation
Olfactory transduction
Ribosome biogenesis in eukaryotes
Spliceosome
Glycosphingolipid biosynthesis − lacto and neolacto series
Ribosome
Other types of O−glycan biosynthesis
Vasopressin−regulated water reabsorption
Biosynthesis of unsaturated fatty acids
Circadian rhythm − mammal
Taste transduction
One carbon pool by folate
Cyanoamino acid metabolism
DNA replication
Mismatch repair
Nucleotide excision repair
Homologous recombination
Non−homologous end−joining
Folate biosynthesis
Pyruvate metabolism
Fatty acid metabolism
SNARE interactions in vesicular transport
Drug metabolism − other enzymes
Propanoate metabolism
Peroxisome
Citrate cycle (TCA cycle)
Glycine, serine and threonine metabolism
Lysine degradation
Renin−angiotensin system
Aminoacyl−tRNA biosynthesis
Basal transcription factors
Oxidative phosphorylation
RNA degradation
Selenocompound metabolism
Mucin type O−Glycan biosynthesis
Vibrio cholerae infection
Gastric acid secretion
mRNA surveillance pathway
Collecting duct acid secretion
Purine metabolism
Pentose phosphate pathway
Glutathione metabolism
Nicotinate and nicotinamide metabolism
Pyrimidine metabolism
PPAR signaling pathway
Arginine and proline metabolism
Protein digestion and absorption
Lysosome
Cardiac muscle contraction
Butanoate metabolism
Salivary secretion
Proximal tubule bicarbonate reclamation
Porphyrin and chlorophyll metabolism
Primary bile acid biosynthesis
Asthma
Steroid hormone biosynthesis
Sulfur metabolism
Primary immunodeficiency
Base excision repair
Intestinal immune network for IgA production
Staphylococcus aureus infection
Complement and coagulation cascades
African trypanosomiasis
Amoebiasis
Malaria
Systemic lupus erythematosus
Proteasome
Regulation of autophagy
Antigen processing and presentation
Autoimmune thyroid disease
Graft−versus−host disease
Type I diabetes mellitus
Allograft rejection
Maturity onset diabetes of the young
Notch signaling pathway
Prion diseases
Basal cell carcinoma
Hedgehog signaling pathway
Metabolic pathways
Cysteine and methionine metabolism
Alzheimer's disease
Parkinson's disease
Hematopoietic cell lineage
Cell adhesion molecules (CAMs)
Viral myocarditis
Phagosome
ECM−receptor interaction
Arrhythmogenic right ventricular cardiomyopathy (ARVC)
Hypertrophic cardiomyopathy (HCM)
Dilated cardiomyopathy
Phosphatidylinositol signaling system
Inositol phosphate metabolism
Type II diabetes mellitus
Aldosterone−regulated sodium reabsorption
Ubiquitin mediated proteolysis
Adipocytokine signaling pathway
Leishmaniasis
NOD−like receptor signaling pathway
RIG−I−like receptor signaling pathway
Cytosolic DNA−sensing pathway
Fc gamma R−mediated phagocytosis
Carbohydrate digestion and absorption
Leukocyte transendothelial migration
Pathogenic Escherichia coli infection
Shigellosis
Bacterial invasion of epithelial cells
Epithelial cell signaling in Helicobacter pylori infection
Huntington's disease
Protein processing in endoplasmic reticulum
Amyotrophic lateral sclerosis (ALS)
Oocyte meiosis
Rheumatoid arthritis
Cytokine−cytokine receptor interaction
Long−term depression
Long−term potentiation
Natural killer cell mediated cytotoxicity
Melanogenesis
Regulation of actin cytoskeleton
Axon guidance
Dorso−ventral axis formation
GnRH signaling pathway
Gap junction
TGF−beta signaling pathway
Endocytosis
Adherens junction
mTOR signaling pathway
VEGF signaling pathway
Renal cell carcinoma
Wnt signaling pathway
Thyroid cancer
Jak−STAT signaling pathway
Acute myeloid leukemia
Neurotrophin signaling pathway
MAPK signaling pathway
Colorectal cancer
Endometrial cancer
ErbB signaling pathway
Hepatitis C
Cell cycle
p53 signaling pathway
Focal adhesion
Small cell lung cancer
Pancreatic cancer
Non−small cell lung cancer
Bladder cancer
Pathways in cancer
Chronic myeloid leukemia
Prostate cancer
Glioma
Melanoma
Tight junction
Progesterone−mediated oocyte maturation
Fc epsilon RI signaling pathway
Insulin signaling pathway
Apoptosis
Toxoplasmosis
Toll−like receptor signaling pathway
Osteoclast differentiation
Chagas disease (American trypanosomiasis)
B cell receptor signaling pathway
Chemokine signaling pathway
T cell receptor signaling pathway
Neuroactive ligand−receptor interaction
Calcium signaling pathway
Vascular smooth muscle contraction
Pancreatic secretion
Vitamin digestion and absorption
Bile secretion
Fat digestion and absorption
ABC transporters
RNA transport
Steroid biosynthesis
Glycerolipid metabolism
Nitrogen metabolism
Alanine, aspartate and glutamate metabolism
Glycosylphosphatidylinositol(GPI)−anchor biosynthesis
Sphingolipid metabolism
Galactose metabolism
Glycolysis / Gluconeogenesis
Fructose and mannose metabolism
Starch and sucrose metabolism
Amino sugar and nucleotide sugar metabolism
Butirosin and neomycin biosynthesis
Glycerophospholipid metabolism
Ether lipid metabolism
alpha−Linolenic acid metabolism
Arachidonic acid metabolism
Metabolism of xenobiotics by cytochrome P450
Drug metabolism − cytochrome P450
Linoleic acid metabolism
Retinol metabolism
Tryptophan metabolism
Histidine metabolism
Tyrosine metabolism
Phenylalanine metabolism

Supplementary table legends

Supplementary Table 1

Data associated to the miRNA signature of Alzheimer's disease (AD-up and AD-down) defined by Satoh 2012(6). Significance (FDR<0.05) is highlighted by red color in cells. Data are structured across different sheets as follows :

Original microRNA: miRNAs as provided in the original publication and their mapping in mirTarBase(5).

mirTarBase Targets: Entrez ID of genes targeted by AD-up and AD-down miRNAs according to mirTarBase.

AD-up S1 Pathways: Enrichment analysis results when applying strategy 1 on AD-up miRNAs. The first 3 columns are related to the significance of association with the original signature. The remaining columns show the distribution of the FDR for each pathway when applying the strategy 1 to 1000 random selections of 16 miRNAs.

AD-down S1 Pathways: Enrichment analysis results when applying strategy 1 on AD-down miRNAs. The first 3 columns are related to the significance of association with the original signature. The remaining columns show the distribution of the FDR for each pathway when applying the strategy 1 to 1000 random selections of 99 miRNAs.

AD-up S2 Pathways: Enrichment analysis results when applying strategy 2 on AD-up miRNAs. The first 3 columns are related to the significance of association with the original signature. The remaining columns show the distribution of the FDR for each pathway when applying the strategy 2 to 1000 random selections of 16 miRNAs.

AD-down S2 Pathways: Enrichment analysis results when applying strategy 2 on AD-down miRNAs. The first 3 columns are related to the significance of association with the original signature. The remaining columns show the distribution of the FDR for each pathway when applying the strategy 2 to 1000 random selections of 99 miRNAs.

AD-down S3 Pathways: Enrichment analysis results when applying strategy 3 on AD-down miRNAs. The first 3 columns are related to the significance of association with the original signature. The last 3 columns indicates to which cluster of Supplementary Figure 2 each pathway belongs, the number of significant pathways (FDR < 0.05)compared to the total number of pathways in the corresponding cluster. The remaining columns show the distribution of the FDR for each pathway when applying the strategy 3 to 1000 random selections of 99 miRNAs.

Supplementary Table 2

All mirTarBase Targets S1 KEGG: KEGG pathways enriched in all protein coding genes targeted by at least one miRNA according to mirTarBase. The first 3 columns show the significance of the association. The remaining columns indicate pathways identified in different studies(6–9) focused on different topics.

Top-Targeted S2 KEGG: KEGG pathways enriched in genes targeted by at least one miRNA, in the top 1000, 500, 100 and 50 genes targeted by miRNAs according to mirTarBase. Only FDR is shown.

Supplementary Table 3

Data associated to the miRNA signature of Chordomas defined by Long *et al.* 2013(7). Significance (FDR<0.05) is highlighted by red color in cells. Data are structured across different sheets as follows :

Original microRNA: miRNAs as provided in the original publication and their mapping in mirTarBase(5).

Chordoma-up S3 Pathways: Enrichment analysis results when applying strategy 3 on Chordoma-up miRNAs. The first 3 columns are related to the significance of association with the original signature. The last 3 columns indicates to which cluster of Supplementary Figure 2 each pathway belongs, the number of significant pathways (FDR < 0.05)compared to the total number of pathways in the corresponding cluster. The remaining columns show the distribution of the FDR for each pathway when applying the strategy 3 to 1000 random selections of 19 miRNAs.

References

1. Fluck, J. (2007) ProMiner: Recognition of human gene and protein names using regularly updated dictionaries. In *Second BioCreative Challenge Evaluation Workshop 2007. Proceedings*.pp. 149–151.

2. Mørk,S., Pletscher-Frankild,S., Palleja Caro,A., Gorodkin,J. and Jensen,L.J. (2013) Protein-driven inference of miRNA-disease associations. *Bioinforma. Oxf. Engl.*, 10.1093/bioinformatics/btt677.

3. Nelson,S.J., Schopen,M., Savage,A.G., Schulman,J.-L. and Arluk,N. (2004) The MeSH translation maintenance system: structure, interface design, and implementation. *Stud. Health Technol. Inform.*, **107**, 67–69.

4. Carlson,M. KEGG.db: A set of annotation maps for KEGG.

5. Hsu,S.-D., Tseng,Y.-T., Shrestha,S., Lin,Y.-L., Khaleel,A., Chou,C.-H., Chu,C.-F., Huang,H.-Y., Lin,C.-M., Ho,S.-Y., *et al.* (2014) miRTarBase update 2014: an information resource for experimentally validated miRNA-target interactions. *Nucleic Acids Res.*, **42**, D78–85.

6. Satoh,J. (2012) Molecular network of microRNA targets in Alzheimer's disease brains. *Exp. Neurol.*, **235**, 436–446.

7. Long,C., Jiang,L., Wei,F., Ma,C., Zhou,H., Yang,S., Liu,X. and Liu,Z. (2013) Integrated miRNA-mRNA analysis revealing the potential roles of miRNAs in chordomas. *PloS One*, **8**, e66676.

8. Ziats,M.N. and Rennert,O.M. (2014) Identification of differentially expressed microRNAs across the developing human brain. *Mol. Psychiatry*, **19**, 848–852.

9. Stather,P.W., Sylvius,N., Wild,J.B., Choke,E., Sayers,R.D. and Bown,M.J. (2013) Differential microRNA expression profiles in peripheral arterial disease. *Circ. Cardiovasc. Genet.*, **6**, 490–497.
